# Integration of Conductive SnO_2_ in Binary Organic Solar Cells with Fine-Tuned Nanostructured D18:L8-BO with Low Energy Loss for Efficient and Stable Structure by Optoelectronic Simulation

**DOI:** 10.3390/nano15050368

**Published:** 2025-02-27

**Authors:** Mohamed El Amine Boudia, Cunlu Zhao

**Affiliations:** Ministry of Education Key Laboratory of Thermo-Fluid Science and Engineering, School of Energy and Power Engineering, Xi’an Jiaotong University, Xi’an 710049, China; amineboudia@stu.xjtu.edu.cn

**Keywords:** binary organic solar cells, SnO_2_, electron transport layer, bulk heterojunction, fine-tuned active layer, photon absorption, power conversion efficiency, thermal stability

## Abstract

Enhancing the performance of organic solar cells (OSCs) is essential for achieving sustainability in energy production. This study presents an innovative strategy that involves fine-tuning the thickness of the bulk heterojunction (BHJ) photoactive layer at the nanoscale to improve efficiency. The organic blend D18:L8-BO is utilized to capture a wide range of photons while addressing the challenge of minimizing optical losses from low-energy photons. The research incorporates SnO_2_ and ZnO as electron transport layers (ETLs), with PMMA functioning as a hole transport layer (HTL). A comprehensive analysis of photon absorption, charge carrier generation, localized energy fluctuations, and thermal stability reveals their critical role in enhancing the efficiency of D18:L8-BO active films. Notably, introducing SnO_2_ as an ETL significantly decreased losses and modified localized energy, achieving an impressive efficiency of 19.85% at an optimized blend thickness of 50 nm with low voltage loss (ΔV_oc_) of 0.4 V within a J_sc_ of 28 mA cm^−2^ by performing an optoelectronic simulation employing “Oghma-Nano 8.1.015” software. In addition, the SnO_2_-based structure conserved 88% of the PCE at 350 K compared to room temperature PCE, which describes the high thermal stability of this structure. These results demonstrate the potential of this methodology in improving the performance of OSCs.

## 1. Introduction

The integration of polymers with non-fullerene acceptors (NFAs) in nanostructured junctions has significantly transformed the performance and architecture of organic semiconductors, especially in the context of OSCs [1,2,3,4,5]. NFAs present notable benefits compared to conventional fullerene-based systems, such as adjustable absorption spectra, superior morphological stability, and improved charge transport characteristics [5,6]. These attributes facilitate more effective exciton dissociation and charge extraction at the donor–acceptor interface, resulting in enhanced PCEs in OSCs.

The relationship between exciton diffusion length and the thickness of bulk heterojunctions (BHJs) is essential for the optimization of organic solar cell efficiency. Additionally, exciton diffusion length is significant across various solar cell types, including perovskite solar cells (PSCs), contributing to the enhancement of device performance [7,8]. In the process of light absorption, excitons are generated as electron–hole pairs that must traverse to the donor–acceptor interface to effectively dissociate into free charge carriers. The exciton diffusion length (L_D_) typically falls between 10 nm and over 40 nm, contingent upon the material characteristics and molecular architecture [9,10]. In a BHJ configuration, it is vital to achieve a precise balance in the active surface thickness; exceeding the exciton diffusion length may lead to a considerable number of excitons recombining before they can reach the interface, thus impairing charge generation efficiency [10,11]. Conversely, if the layer is too thin, it may not fully exploit the light absorption potential of the active materials. Therefore, an optimal thickness is necessary to ensure that excitons can effectively reach the interface while maximizing photon absorption. Investigations have demonstrated that adjusting the morphology and domain sizes within BHJs can enhance exciton diffusion, thereby improving the overall performance of the device [6]. This dynamic emphasizes the importance of designing BHJ layers that are compatible with exciton diffusion characteristics to achieve efficient carrier separation and collection in OSCs [6].

The enhancement of PCE in single-junction OSCs significantly relies on the incorporation of advanced materials and the deliberate arrangement of donor and acceptor components [11,12]. Recent research has shown that the application of advanced NFAs in conjunction with novel donor polymers can markedly enhance device performance [13]. For example, a methodical strategy that includes the design and virtual evaluation of donor–acceptor pairs has resulted in the discovery of new molecular combinations, highlighting the promise of high-efficiency organic photovoltaics [14,15,16].

D18, a copolymer with a narrow bandgap, has proven to be an exceptionally effective material for organic solar cells, owing to its remarkable characteristics [17,18]. Its structure features a backbone that alternates between electron-donating benzodithiophene (BDT) units and electron-accepting fused-ring dithienobenzothiadiazole (DTBT) units, resulting in a high level of conjugation that significantly improves charge transport efficiency [19]. Recent research indicates that devices employing D18 as the electron donor, especially when paired with NFAs such as Y6, can achieve notable PCEs surpassing 18% [19]. For example, one investigation reported a certified efficiency of 17.6%, with a short-circuit current density (Jsc) of 27.7 mA/cm^2^ and an open-circuit voltage (V_oc_) of 0.859 V for the D18:Y6 blend. The polymer also demonstrates a high hole mobility, estimated at around 1.59 × 10^−7^ m^2^ V^−1^ s^−1^.

A recent investigation into the D18:L8-BO OSC, utilizing double-phenanthroline-carbolong, achieved a remarkable efficiency of 18.2%. The J_sc_ measured was 24.64 mA cm^−2^, with a V_oc_ of 0.89 V, highlighting the effective charge generation and transport properties of the D18:L8-BO blend [20]. This notable performance is attributed to the optimized morphology and improved intermolecular interactions resulting from the introduction of innovative non-fullerene acceptors, which enhanced exciton dissociation and minimized charge recombination losses.

The careful selection of interface materials, particularly the ETL and HTL, plays a vital role in improving the efficiency of OSCs. Recent research highlights that the appropriate selection of ETL and HTL materials can facilitate more efficient pathways for charge carriers, which in turn reduces energy offsets and enhances mobility [21,22].

Poly(methyl 2-methylpropenoate) (PMMA) is a prominent thermoplastic polymer recognized for its remarkable optical transparency, resistance to ultraviolet light, and durability against environmental factors, rendering it a valuable material in the realm of OSCs [23]. Its characteristics, which include substantial transparency, robust mechanical properties, and minimal moisture permeability, play a crucial role in improving the stability and efficiency of these devices. In the context of OSCs, PMMA is frequently employed as an HTL or integrated into hybrid configurations to enhance charge transport and mitigate recombination losses. The use of PMMA can optimize the interface between the active layer and the electrodes, resulting in improved energy level alignment and increased charge mobility [24,25,26].

A significant investigation involving PMMA was performed on inverted OSCs utilizing a PMMA-doped nickel oxide (NiO) HTL [27]. This study achieved a PCE of 15.11% alongside a V_oc_ of 0.90 V. The incorporation of PMMA enhanced the electrical characteristics of the HTL and promoted more effective charge carrier transport, which was instrumental in attaining the elevated V_oc_ observed in the devices [27]. This research underscores the capability of PMMA to not only boost device performance but also to ensure stability in ambient conditions.

Zinc oxide (ZnO) serves as a prominent ETL OSC owing to its beneficial characteristics [28,29,30]. As an n-type semiconductor, ZnO is characterized by its excellent electron mobility, durability, and optical transparency [28]. A significant investigation utilizing ZnO as an ETL demonstrated remarkable outcomes when paired with the D18:N3 active layer, resulting in a substantial V_oc_ of 0.90 V and an FF of 0.75, which culminated in a PCE of 18.20%. This study underscored the role of ZnO in improving charge extraction and transport, which ultimately facilitated the elevated V_oc_ and FF observed in solar cell devices [31].

Tin dioxide (SnO_2_) possesses several significant characteristics that render it an advantageous material for application in OSCs as an ETL [31,32]. With a high melting point exceeding 1630 °C, a density of 6.95 g cm^−3^, and remarkable thermal stability [32], SnO_2_ is well-suited for maintaining stable device performance across diverse operational conditions [33,34]. Its optimal refractive index of 1.83 ensures substantial optical transparency, facilitating efficient light transmission. In parallel, both SnO_2_ and titanium dioxide (TiO_2_) are inorganic semiconducting metal oxides utilized as electron transport layers [35,36], yet SnO_2_ offers distinct advantages over TiO_2_. Characterized as an n-type semiconductor, SnO_2_ possesses a wide band gap of 3.6 eV, along with notable optical transparency and low resistivity [37]. The electron mobility in SnO_2_ ranges from 100 to 200 cm^2^ V^−1^ s^−1^, suggesting a more rapid transport of photo-excited electrons. Additionally, the conduction band minimum (CBM) of SnO_2_ is approximately 0.4 eV lower than that of TiO_2_, thereby enhancing electron transport efficiency [37,38]. Empirical studies have shown that SnO_2_/MAPbI3 heterojunction interfaces exhibit a greater capacity for electrical transport and are less susceptible to electron trapping compared to their TiO_2_/MAPbI3 counterparts [39]. The increased electron concentration in SnO_2_ is primarily due to oxygen vacancies, which significantly enhance its electron transport capabilities. Furthermore, SnO_2_ has shown significant performance enhancement in some theoretical studies for ternary OSCs [18,40].

In its role as an ETL, SnO_2_ aids in aligning energy levels between the active layer and the electrode, thereby enhancing electron extraction and transport while reducing recombination losses [34,41].

In this study, we performed an optoelectronic simulation of binary OSC composed of D18:L8-BO (1:1.2) as a nanostructured active blend with an optimized thickness of 50 nm employing PMMA as an HTL and SnO_2_ and ZnO as the ETLs. This was achieved using the drift-diffusion theory, working with an organic and hybrid material nano one-two dimension optical, electrical, and thermal simulation software tool (Oghma-Nano 8.1.015). In addition, we fine-tuned the active blend within 50–200 nm to optimize the efficiency of OSCs in terms of photon absorption, exciton generation, and free charge carriers generation and transport toward the anode and cathode contacts. Furthermore, we selected ETLs such as SnO_2_ and ZnO due to their impressive optoelectronic properties (as investigated in our previous studies) in terms of electron transport, high resistivity to prevent charge recombination, structural thermal stability, and resistance to moisture and temperature. In parallel, we investigated the thermal stability of SnO_2_-based devices, increasing temperature within the range of 300–400 K and keeping an eye on their performance behavior in terms of efficiency parameters. We have conducted these investigations and simulations to obtain results on efficient and stable binary OSC. This simulation is validated from a study conducted by Zhu et al. [42], where we modified the selection of the charges transport layers and fine-tuned the thickness of the active blend by theoretical simulation. Our study provides insights into employing novel interface materials in binary OSCs to obtain high efficiency and stability in single-junction OSCs.

## 2. Method

**Figure 1:**
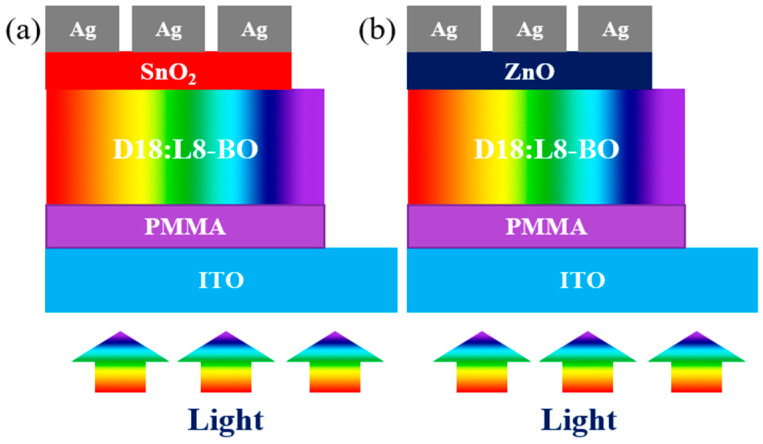
(**a**) SnO_2_-based binary OSC under light exposure; (**b**) ZnO-based binary OSC under light exposure.

**Figure 2:**
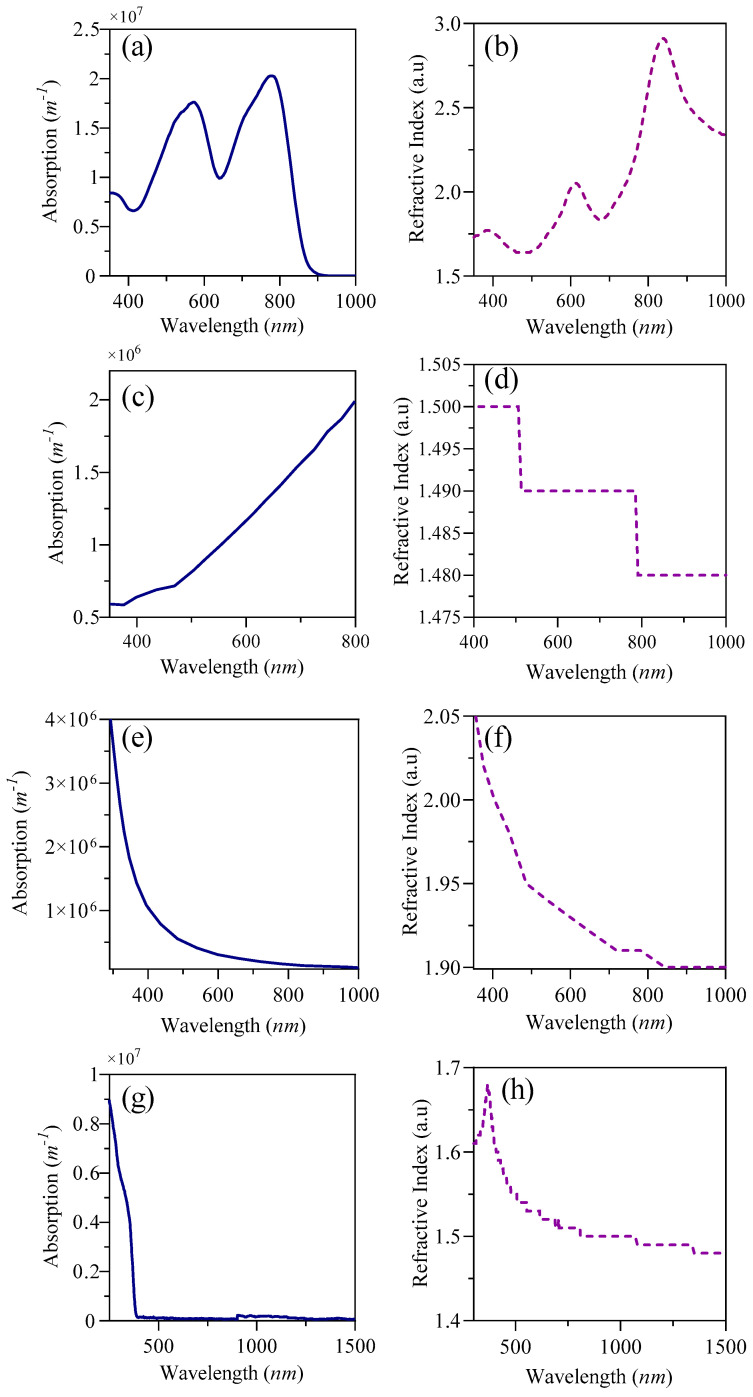
(**a**) D18:L8-BO absorption rate; (**b**) D18:L8-BO refractive index; (**c**) PMMA absorption rate; (**d**) PMMA refractive index; (**e**) SnO_2_ absorption rate; (**f**) SnO_2_ refractive index; (**g**) ZnO absorption rate; and (**h**) ZnO refractive index.

The optoelectronic simulation employs “Oghma-Nano 8.1.015”, specifically developed to resolve optical and electrical equations to derive performance results. The electrical model encompasses Poisson’s equation, the statistical behavior of free charge carriers, and the drift-diffusion equations governing charge carrier flow. Conversely, the optical model is constructed upon the Maxwell equations as articulated within the frequency domain. The device’s architecture consists of Indium Tin Oxide (ITO)/PMMA/D18:L8-BO (1:1.2)/ETL/Silver (Ag) as mentioned in Figure 1a,b within a device area of 4.84 mm^2^. The materials thicknesses and the detailed input parameters for optical and electrical aspects are provided in the accompanying Figure 2 and Table 1, Table 2 and Table 3.

The Oghma-Nano tool was developed to address the optical, electrical, and thermal modeling of optoelectronic devices, specifically within OSCs, across various domains, including time, steady state, and frequency. The methodology incorporates a finite difference approach to resolve a spectrum of equations, encompassing the frequency equation pertinent to Maxwell’s equations, the absorbed energy density in the active layer, drift-diffusion equations, charge carrier continuity equations, and Poisson’s equation [43]. In the optical model, the frequency equation from Maxwell’s domain is utilized in a one-dimensional framework to determine the photon distribution, with the electromagnetic fields described by Equations (1) and (2),

(1) $$\frac{\partial \vec{H_{opt}}}{\partial x}=j2\pi \nu \varepsilon_{r}\varepsilon_{0}\vec{E_{opt}}$$

(2) $$\frac{\partial \vec{E_{opt}}}{\partial x}=-j2\pi \nu {µ}_{0}\vec{H_{opt}}$$

where $\vec{H_{opt}}$ is the vector of the magnetic field, $\vec{E_{opt}}$ is the vector of the electric field, ν is the frequency, and ε_0_ and εr are the free and relative permittivity, respectively.

The absorbed energy denotes the quantity of electromagnetic energy the active layer material captures at a specific point along the axis [43,44]. The spectral absorbed energy density is as follows in Equations (3) and (4):

(3) $$Q\left(\nu ,x\right)=\frac{1}{2}c\varepsilon_{0}\alpha \eta E_{opt}^{2}$$

(4) $$\alpha =\frac{2\pi k}{\lambda }$$

In this context, c represents the speed of light, η denotes the refractive index, α signifies the absorption coefficient, and λ indicates the wavelength of the incident light. The emergence of excitons was influenced by the photon absorption, culminating in the production of a specific amount, as evidenced by Equation (5) for each frequency and spatial area [44,45].

(5) $$G\left(\nu ,x\right)=\frac{Q}{h\nu }=\frac{\pi \varepsilon_{r}\varepsilon_{0}}{h}E_{opt}^{2}$$

where h is Planck’s constant. The carrier continuity equations are fundamental in the field of semiconductor physics, as they clarify the behavior of charge carriers, specifically electrons and holes, within a given material. These equations account for the processes of generation, recombination, and transport of carriers, thereby maintaining charge conservation within a defined volume [45]. The continuity equations can be expressed in terms of the time-dependent changes in carrier density, while also considering the effects of drift and diffusion currents, in addition to generation and recombination processes. The conventional formulation of the continuity equation for a particular type of carrier, whether it be electrons or holes, is presented as outlined in Equations (6) and (7), which guarantees the conservation of charge carriers [45]:

(6) $$\frac{\partial J_{n}}{\partial x}=q(R_{n}-G+\frac{\partial n}{\partial t})$$

(7) $$\frac{\partial J_{p}}{\partial x}=-q(\;R_{p}-G+\frac{\partial p}{\partial t})$$

Here, R_n_ and R_p_ are defined as the recombination rates for electrons and holes, respectively. The current densities for electrons and holes are represented by J_n_ and J_p_, respectively. In addition, q stands for the charge. Poisson’s equation represents a fundamental partial differential equation within mathematical physics, demonstrating the relationship between a scalar potential field and the distribution of sources, such as charge density in electrostatics or mass density in gravitational scenarios [31,43,45]. Its applications are widespread across various fields, including electrostatics, fluid dynamics, and heat conduction. The device’s potential distribution is obtained by solving Poisson’s equation, and is depicted in Equation (8):

(8) $$\frac{\partial^{2}\varphi }{\partial x^{2}}{ℇ}_{0}{ℇ}_{r}=q(n-p)$$

The drift-diffusion equations are fundamental to modeling charge transport in organic solar cells (OSCs). This model combines the effects of drift, which occurs due to electric fields, and diffusion, which arises from concentration gradients [43]. In OSCs, the application of an electric field generates a force on charge carriers that is directly proportional to their charge, causing them to move toward opposite electrodes and thus enabling current flow. Concurrently, charge carriers also migrate in response to concentration gradients, as described by Fick’s law [44]. This diffusion process is vital for achieving equilibrium in carrier concentrations across different regions of the device, especially in areas where excitons dissociate into free carriers [44]. The charge transfer processes, as described in Equations (9) and (10) below, can be clarified through the resolution of the drift-diffusion equations, which incorporate the dynamics of both electrons and holes:

(9) $$J_{n}=q\mu_{e}n\frac{\partial \varphi }{\partial x}+qD_{n}\frac{\partial n}{\partial x}$$

(10) $$J_{p}=q\mu_{h}p\frac{\partial \varphi }{\partial x}+qD_{p}\frac{\partial p}{\partial x}$$

In this context, J_n,p_ denotes the current density for electrons and holes. The symbols μ_e,h_ represent the mobilities of electrons and holes, respectively, while D_n,p_ refers to the diffusion coefficients for electrons and holes, respectively. Tunneling mechanisms for electrons and holes through heterojunction interfaces are utilized to characterize the boundary conditions, as expressed in Equations (11) and (12) [44].

(11) $$J_{n}=-qT_{e}[\left(n_{1}-n_{1}^{eq}\right)-\left(n_{0}-n_{0}^{eq}\right)]$$

(12) $$J_{p}=-qT_{h}[\left(p_{1}-p_{1}^{eq}\right)-\left(p_{0}-p_{0}^{eq}\right)]$$

In this framework, T_h_ and T_e_ are indicative of the tunneling activities of holes and electrons, respectively. The parameters n_0_ and n_1_ correspond to the quantity of electrons found in the different layers, while p_0_ and p_1_ reflect the number of holes in those layers, $n_{0,1}^{eq}$ is the equilibrium number of electrons in the layers, and $p_{0,1}^{eq}$ is the equilibrium number of holes in the layers.

This study aims to advance the PCE of binary OSCs through the implementation of an optimal structural configuration that reduces energy loss and promotes improved efficiency and stability [46]. The performance indicator PCE is represented by Equation (13):

(13) $$\eta =\frac{J_{sc}V_{oc}}{I_{in}}$$

P_max_ represents the peak power output of the OSC, while I_in_ indicates the intensity of illumination. The V_oc_ value signifies the highest voltage recorded in the absence of current flow through the device, and can be articulated using the following equation:

(14) $$Voc=\frac{1}{q}\{E_{g}-2(E_{F,h}-E_{HOMO}^{D})+K_{B}Tln\left(\frac{{µ}_{e}\;N_{c}\frac{\partial \;E_{F,e}}{\partial x}\;}{{µ}_{h}N_{v}\frac{\partial \;E_{F,h}}{\partial x}}\right)\}$$

with $N_{c}=N_{v}$ and $\nabla E_{F,e}=\nabla E_{F,h}$.

As a result: $qV_{oc}=E_{g}-\Delta E$; where,

(15) $$\Delta E=2\left(E_{F,h}-E_{HOMO}^{D}\right)-K_{B}Tln\left(\frac{{µ}_{e}\;}{{µ}_{h}}\right)$$

In these equations, q is the charge; K_B_ is the Boltzmann constant; T is the temperature; µe and µh are the electron and hole mobilities, respectively; and N_c_ and N_v_ are the conduction and valence bands effective densities of the states, respectively.

The J_sc_ in OSCs is predominantly determined by the light absorption within the active layer and the rate at which charges are generated [47]. The fundamental equation that describes J_sc_ can be articulated as:

(16) $$J_{sc}=q\;\int_{0}^{\infty }\varphi \left(E\right)\;\eta \left(E\right)dE$$

where φ is the photon flux at energy E. The FF can be calculated by utilizing the subsequent Equation (17).

(17) $$FF=\frac{P_{max}}{V_{oc}J_{sc}}$$

Detailed information regarding the optoelectronic model employed in our simulation is available in the cited references [43,44,45].

## 3. Results and Discussion

**Figure 3:**
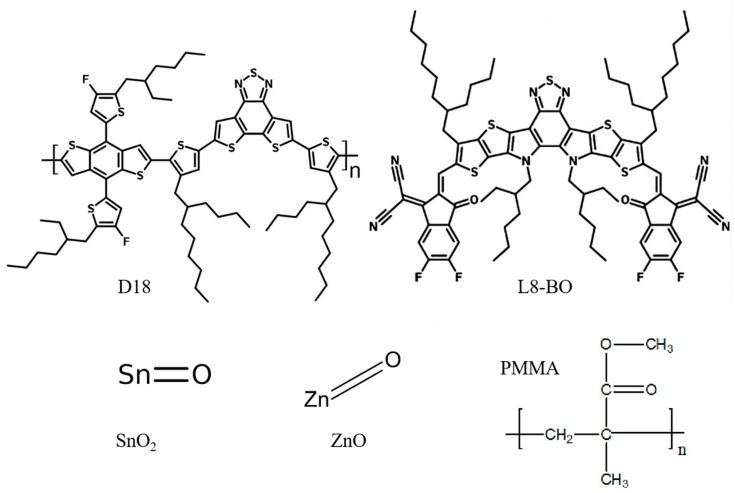
The chemical structures of the materials of the devices.

The chemical structures, absorption wavelengths, and refractive indices of the materials utilized in SnO_2_- and ZnO-based devices are illustrated in Figure 2 and Figure 3. These parameters are essential for determining the spectral absorbed energy, as outlined in equation 3, to achieve a more precise solution of the optoelectronic model, ultimately leading to enhanced performance outcomes. The D18:L8-BO active layer mixture primarily absorbs photons within the range of 350 to 900 nm, with D18 specifically capturing photons with shorter wavelengths from 400 to 700 nm [17]. This mixture also aids in controlling the crystallization processes of the materials, resulting in a donor phase with superior crystalline characteristics [42]. PMMA absorbs photons from 340 to 800 nm, encompassing the entire visible spectrum, and possesses a refractive index of 1.49, as indicated in Figure 2d, which facilitates the absorption of visible wavelengths from the active layer. SnO_2_ exhibits minimal absorption in the visible spectrum, which can be advantageous for allowing light to penetrate the active layers of the solar cell. Nonetheless, it does absorb some UV light, particularly in the 300–400 nm range, which can enhance stability under UV exposure, although it does not significantly contribute to energy conversion. ZnO features a wide bandgap (3.1–3.3 eV), restricting its absorption mainly to the UV region; however, when combined with organic materials or semiconductors with smaller bandgaps, its absorption can extend into the visible spectrum. Additionally, PMMA is recognized as an effective material for photon absorption in the visible range and is highly crystalline, serving as an HTL. The careful selection of either a p-i-n or n-i-p structure significantly influences the overall efficiency of the device, particularly concerning photon absorption and charge carrier transport.

**Figure 4:**
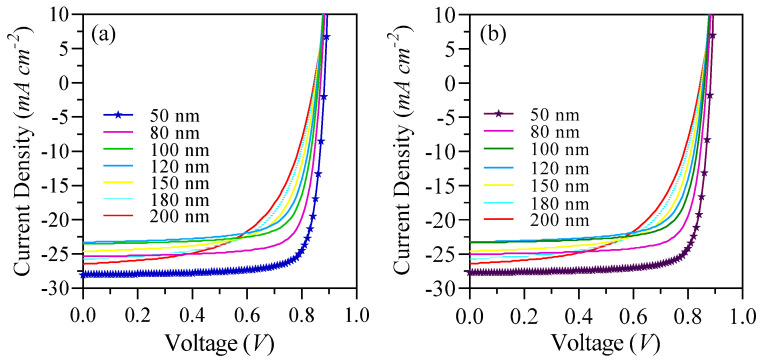
(**a**) Simulated J-V characteristics of the SiO2-based device, (**b**) simulated J-V characteristics of the ZnO-based device.

The simulation of the J-V characteristics density was conducted under AM 1.5 G illumination, with an intensity set at 100 mW cm^−2^. The findings are illustrated in Figure 4a,b and summarized in Table 4. Our devices are composed of S1: ”ITO/PMMA/D18:L8-BO/SnO_2_/Ag”, and S2: “ITO/PMMA/D18:L8-BO/ZnO/Ag”, resulting in an active layer thickness of 50 nm, which is the optimized active layer thickness of champion device PCEs of 19.85% and 19.6%, respectively. Additionally, S1 delivers a V_oc_ of 0.88 V, J_sc_ of 28.2 mA cm^−2^, and an FF of 80%. On the other hand, S2 delivers a V_oc_ of 0.88 V, J_sc_ of 27.7 mA cm^−2^, and an FF of 80.

**Figure 5:**
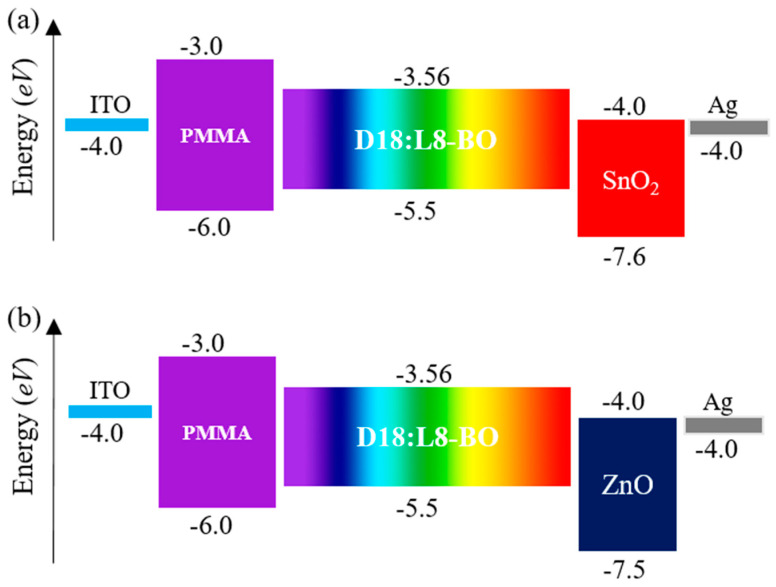
(**a**) Energy levels of S1 materials, and (**b**) energy levels of S2 materials.

The thickening of the active layer was associated with greater light absorption and exciton generation in all devices investigated. However, this advancement necessitates that charge carriers travel a longer distance to reach the electrodes, arising from the inherent constraints of exciton diffusion length characteristic of organic semiconductor materials [48]. This phenomenon may result in heightened bimolecular recombination, consequently reducing the J_sc_. When we examine the impact of varying active layer thicknesses on our PCE parameters, as presented in Table 4, it becomes evident that both the V_oc_ and FF experienced a decline as the active blend thickness increased from 50 nm to 200 nm. Specifically, the V_oc_ decreased from 0.88 V to 0.845 V for both S1 and S2 within this range, while the FF saw a significant reduction from 80% to 59% for S1 and from 80% to 58.6% for S2. In contrast, the J_sc_ exhibited an increase from 28.2 mA cm^−2^ at 50 nm to 23.28 mA cm^−2^ at 120 nm, followed by a slight increase as the thickness continued to rise, ultimately reaching a J_sc_ of 26.4 mA cm^−2^ at 200 nm for S1. The decline in J_sc_ between 50 nm and 120 nm can be caused by the increased distance that excitons must diffuse to reach the lowest unoccupied molecular orbital (LUMO) and highest occupied molecular orbital (HOMO) levels, compounded by the relatively short exciton diffusion length of the organic semiconductors. Notably, the J_sc_ increased again from 120 nm to 200 nm due to the enhanced photon absorption wavelength, which generates a greater number of excitons, despite the challenges posed by the longer travel distance that impedes the separation and dissociation of excitons into free carriers. The reductions in V_oc_ and FF that have been recorded might be associated with multiple factors, including the greater distances that charge carriers must cover to arrive at the interfaces, in addition to an increase in the energy offset (ΔE), which intensifies the challenges faced. Basically, we selected aligned and cascaded energy levels between the selected materials in the S1 and S2 as mentioned in Figure 5a,b. Furthermore, D18:L8-Bo exhibited −5.5 eV and −3.56 eV for HOMO and LUMO, respectively; PMMA exhibited −6.0 eV and −3.0 eV for HOMO and LUMO, respectively; SnO_2_ released −7.6 eV and −4.0 eV for HOMO and LUMO, respectively; and ZnO released −7.5 eV and −4.0 eV for HOMO and LUMO, respectively. We noticed that the energy levels of the different materials are aligned, which contributes to more straightforward pathways, which resulted in high V_oc_ and FF results in our study.

In addition, the intrinsic short exciton diffusion length found in organic semiconductors results in an increase in recombination losses [10]. The dissociation of charge carriers within the organic active blend is limited by their short diffusion length. Consequently, it is essential to achieve an optimal equilibrium between a wide absorption spectrum that promotes exciton generation and a reduced distance for charge carriers to effectively dissociate and migrate toward the electrodes [10].

By employing the optimized thickness of 50 nm for S1, a substantial balance is attained between the increase in absorption and the diffusion of excitons. Consequently, this thickness is identified as the optimal operational point for our device. Additionally, at this thickness, we recorded the highest J_sc_ of 28.2 mA cm^−2^, a V_oc_ of 0.88 V, and an FF of 80%, which collectively represent the best performance among various thicknesses for S1. A similar trend was observed in S2 devices; however, a slight reduction in J_sc_ compared to S1 was noted. The role of the ETL in promoting efficient charge transport is crucial, as it reduces interface resistance and fosters improved alignment of the energy level with the active layer. This synergy enhances exciton dissociation and charge collection efficiency, leading to an increase in J_sc_ values. The minor decrease in J_sc_ for S2 can be linked to the crystalline nature and chemical structure of SnO_2_, which features a tetrahedral configuration where each silicon atom is covalently bonded to four oxygen atoms, forming a three-dimensional silicon-oxygen bond network as depicted in Figure 3. Nonetheless, both SnO_2_ and ZnO are effective materials for use as an ETL, as evidenced by the results obtained.

**Figure 6:**
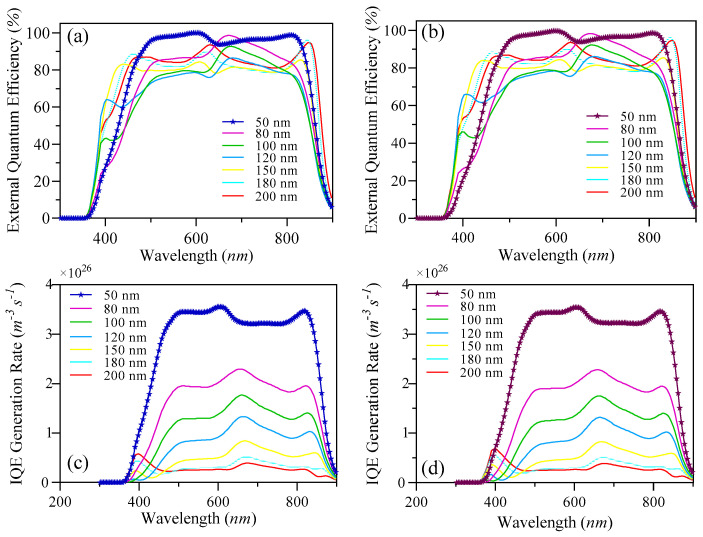
(**a**) The simulated EQE results of S1, (**b**) simulated EQE results of S2, (**c**) simulated IQE results of S1, and (**d**) simulated IQE results of S2.

**Figure 7:**
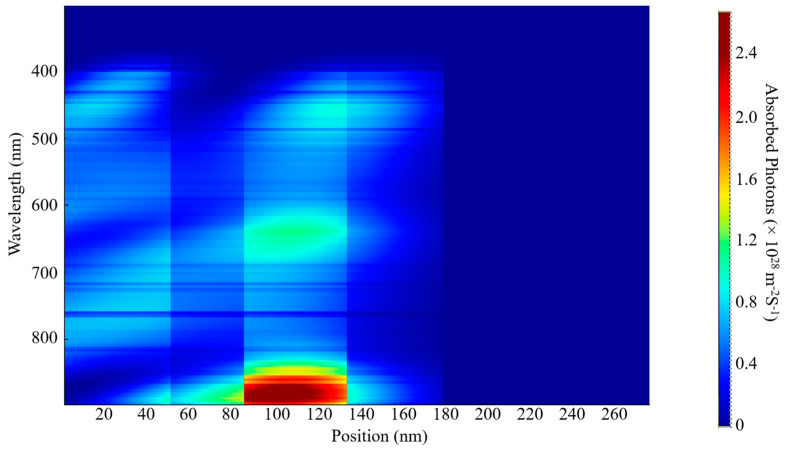
Incident photon distribution of S1.

**Figure 8:**
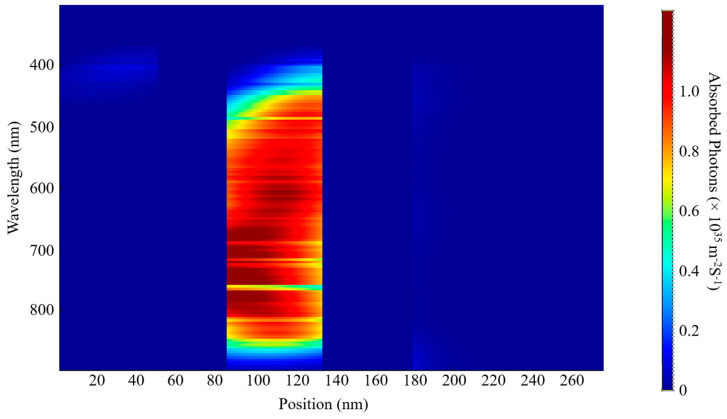
Absorbed photon distribution of S1.

The EQE and IQE outcomes for S1 and S2 are shown in Figure 6. The examination indicates a wide absorption wavelength spectrum spanning from 380 nm to 900 nm for both samples, as shown in Figure 7 and Figure 8. This considerable absorption range plays a crucial role in enhancing the exciton generation rate, as demonstrated in Figure 6c,d. In addition, the D18 donor in this material configuration exhibits excellent crystallinity, which enhances the diffusion and dissociation processes of excitons, thereby contributing to an increase in the J_sc_.

Numerous elements must be considered when optimizing the active layer’s thickness, such as the material’s light-scattering characteristics, charge mobility, absorption coefficient, and exciton diffusion length. This necessitates a careful balance between enhancing photon absorption and reducing charge recombination to determine the optimal thickness for the active blend. In our examination of external quantum efficiency (EQE) and internal quantum efficiency (IQE), we noticed that the highest absorption rates for both S1 and S2 occurred at this specific thickness. These findings can be linked to the effective alignment of absorption wavelengths with this thickness, alongside the efficient diffusion, dissociation, and extraction of free charge carriers. Additionally, a notable exciton generation was recorded, attributed to the advantageous exciton diffusion length. Specifically, the peak generation rates for S1 were measured at 3.6 × 10^26^ m^−3^s^−1^ and 3.5 × 10^26^ m^−3^s^−1^ at wavelengths of 622 nm and 830 nm, respectively. The IQE and EQE values for S2 were found to be approximately comparable to those of S1.

A decrease in the active layer’s thickness results in a shorter pathway for charge carriers to traverse in order to reach the ETL, HTL, and, ultimately, the electrodes. Our study has shown that a film with a thickness of 200 nm presents a wide absorption spectrum, although it is associated with a relatively low charge generation rate. The highest charge generation rates for S1 were recorded at 6.2 × 10^25^ m^−3^ s^−1^ and 4.4 × 10^25^ m^−3^ s^−1^ at wavelengths of 398 nm and 685 nm, respectively. By comparing the two thicknesses, we can confirm the previously established factors that influence the fine-tuning of the thickness of the blend. Furthermore, this research provides insights into achieving the most effective balance between light absorption and the reduction in charge recombination, while also addressing the role of exciton diffusion length.

**Figure 9:**
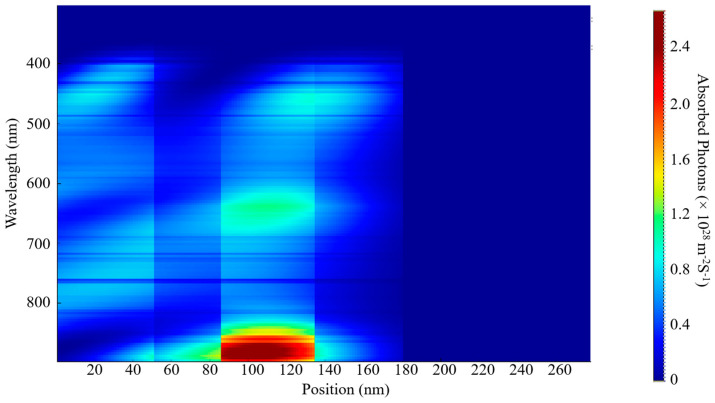
Incident photon distribution S2.

**Figure 10:**
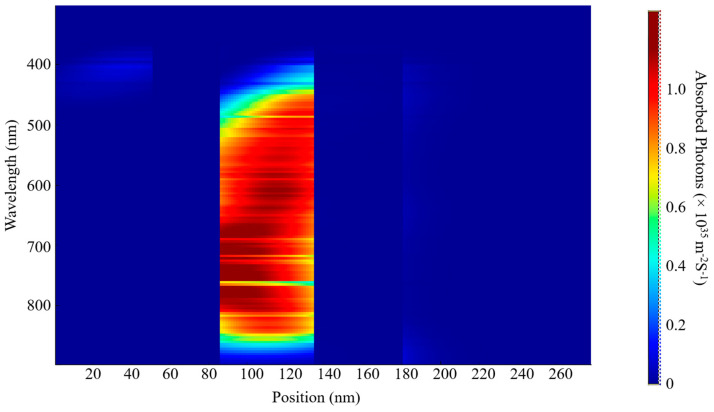
Absorbed photon distribution of S2.

The determination of these parameters is significantly influenced by a variety of factors, including photon absorption, exciton generation and diffusion, their subsequent dissociation, the transport mechanisms at play, and the final collection of free carriers by the electrodes. The spectra of incident and absorbed photons for S1 and S2 are depicted in Figure 7, Figure 8, Figure 9 and Figure 10. The EQE curves presented in Figure 6a,b indicate a substantial degree of photon absorption within the wavelength range of 450 nm to 870 nm. The significance of this range is underscored by a substantial concentration of photon density identified within the spectrum of 350 nm to 880 nm. With an optimal active layer thickness of 50 nm, the light absorption reaches approximately 90%, resulting in an average generation rate of 3.4 × 10^2^⁶ m^−3^ s^−1^ for both S1 and S2. Additionally, a pronounced photon absorption rate is evident in the range of 85 to 135 nm, as shown in Figure 8 and Figure 10. The observed decline in efficiency within the current generation of thicker devices may stem from the limited exciton diffusion length. Although these materials possess robust light absorption properties, they face significant challenges in the effective separation of electrons and holes, largely due to diffusion constraints, as previously analyzed.

**Figure 11:**
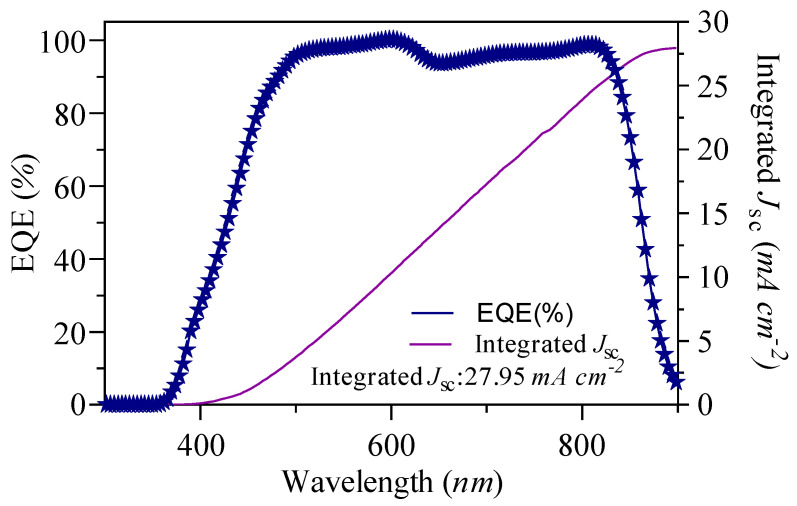
The integrated J_sc_ and EQE of SnO_2_-based device at optimal thickness of 50 nm.

The total current produced by OSC is intrinsically related to its EQE. By evaluating the EQE of the cell throughout the complete solar electromagnetic spectrum, one can ascertain the current output when the cell is subjected to sunlight [49]. The findings regarding the integrated current are illustrated in Figure 11 for sample S1. The correlation between EQE(λ) and current is directly proportional to the current per unit of photon flux. The J_sc_ obtained from the J-V curve aligns well with the integrated J_sc_ derived from the EQE, measuring 28 mA cm^−2^, as depicted in Figure 11.

The Shockley–Queisser (SQ) limit signifies the theoretical maximum efficiency that can be attained by a single p-n junction solar cell, a threshold established by fundamental energy loss mechanisms that are intrinsic to the energy conversion process [50]. One of the key loss mechanisms is thermalization, which occurs when photons with energy greater than the semiconductor’s bandgap create electron–hole pairs, with the excess energy being lost as heat rather than being utilized for electricity generation [51]. Another critical aspect is spectral mismatch; not every photon in the solar spectrum has sufficient energy to generate electron–hole pairs, and the energy from those that do, which greatly exceeds the bandgap, is wasted as heat [51,52]. Additionally, radiative recombination contributes to energy losses, as it involves the direct recombination of electrons and holes, resulting in the release of energy as photons instead of contributing to the electrical current, thus limiting the open-circuit voltage [51]. Parasitic resistance, which includes resistance within the semiconductor, contacts, and external circuits, further reduces efficiency by dissipating energy as heat. These combined factors impose a theoretical limit on the performance of single-junction solar cells, highlighting the challenges associated with achieving higher efficiencies [53].

The Shockley–Queisser limit establishes a theoretical upper bound on the efficiency of single-junction solar cells, which is approximately 33.7% for materials with an optimal bandgap of about 1.34 eV when evaluated under standard test conditions (AM 1.5G) [50]. The analysis of calculated losses reveals the impact of different mechanisms on the total voltage output of solar cells, underscoring the necessity of reducing these losses to improve overall efficiency [50].

The J_sc_ is given by Equation (18),

(18) $$J_{sc}=q\int_{\lambda_{1}}^{\lambda_{2}}\frac{EQE\left(\lambda \right)\;AM\;1.5G\;\left(\lambda \right)\;d(\lambda )}{\frac{hc}{\lambda }}$$

V_oc_ for Shockley–Quisser (S–Q) limit is given by Equation (19),

(19) $$V_{oc}^{SQ}=\frac{kT}{q}\ln\left(\frac{J_{sc}^{SQ}}{J_{0}^{SQ}}+1\right)=\frac{KT}{q}\ln\left(\frac{\int_{E_{g}}^{\infty }\varphi_{AM1.5G}\left(E\right)\;d(E)}{\int_{E_{g}}^{\infty }\varphi_{BB}\left(E\right)\;d(E)}\right)$$

V_oc_ for radiative recombination limit is given by Equation (20).

(20) $$V_{oc}^{rad}=\frac{KT}{q}\ln\left(\frac{J_{sc}}{J_{0}^{rad}}+1\right)=\frac{KT}{q}\ln\left(\frac{\int_{-\infty }^{\infty }{EQE}_{AM1.5G}\left(E\right)\;d(E)}{\int_{-\infty }^{\infty }{EQE\left(E\right)\;\varphi }_{BB}\left(E\right)\;d(E)}\right)$$

Total voltage losses (ΔV_oc_) are given by Equation (21),

(21) $$∆V_{oc}=\frac{E_{g}}{q}-V_{oc}=\left(\;\frac{E_{g}}{q}-V_{oc}^{SQ}\right)+\left(V_{oc}^{SQ}-V_{oc}^{rad}\right)+\left(V_{oc}^{rad}-V_{oc}\right)=∆V_{1}+∆V_{2}+∆V_{3}\;\;Shockley-Queisserlimit\left(V_{oc}^{SQ}\right)=1.0651\;V\;Radiadive\;limit\;\left(V_{oc}^{rad}\right)=1.155\;V\;∆V_{1}=\frac{E_{g}}{q}-V_{oc}^{SQ}=0.2549\;V\;∆V_{2}=Radiative\;loss\;\left(V_{oc}^{SQ}-V_{oc}^{rad}\right)=-0.0899\;V\;∆V_{3}=Non-radiativeloss\;\left(V_{oc}^{rad}-V_{oc}\right)=0.2728\;V\;\Delta V_{oc}=Total\;loss\left(\frac{E_{g}}{q}-V_{oc}\right)=0.4378\;V$$

ΔV1 represents the thermodynamic loss associated with the energy required to transition electrons from the valence band to the conduction band. This loss can also be characterized as the disparity between the bandgap energy and the thermodynamic open-circuit voltage (q$V_{oc}^{SQ}$). Generally, ΔV signifies the radiative recombination that occurs above the optical gap, a phenomenon that is inherent to all solar cells [51].

ΔV2 represents the radiative loss, defined as the discrepancy between the Shockley–Queisser limit for open-circuit voltage ($V_{oc}^{SQ}$) and the radiative limit for open-circuit voltage ($V_{oc}^{rad}$). This loss is a consequence of radiative recombination occurring above the optical gap, a phenomenon that is inherent to the operation of solar cells [51].

ΔV3 signifies the non-radiative energy loss in solar cells, which encompasses energy dissipation via processes that do not involve light emission. This includes interactions with phonons, where energy is converted into heat. Minimizing ΔV3 can enhance the efficiency of solar cells [52].

**Figure 12:**
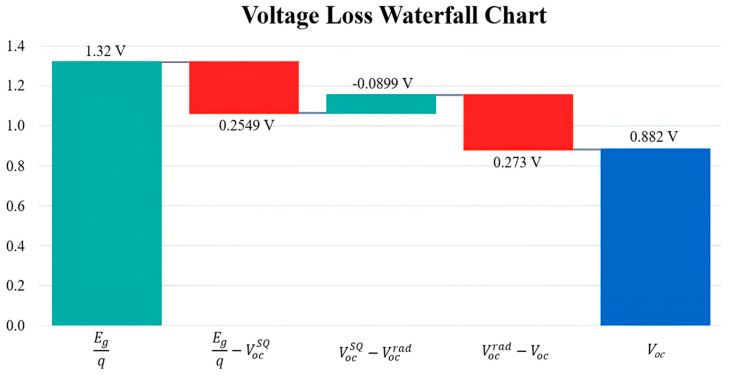
The voltage loss diagram for S1 device at 50 nm thickness.

The calculated values for both the S-Q limit and the radiative limit indicate that the performance of the SiO_2_-based device is in close alignment with theoretical predictions. ΔV_1_ demonstrates an acceptable reduction from the bandgap energy to the S-Q limit, which aligns with expectations. ΔV_2_ reveals a minor radiative loss, which could be reduced through enhanced design and material selection. ΔV_3_ illustrates non-radiative losses; a positive value implies that considerable non-radiative recombination processes are taking place, presenting an opportunity for further enhancement. The overall loss of 0.4378 V suggests that while the OSC operates effectively, there remain opportunities for optimization based on the calculated results of the S-Q limit and the data presented in Figure 12.

**Figure 13:**
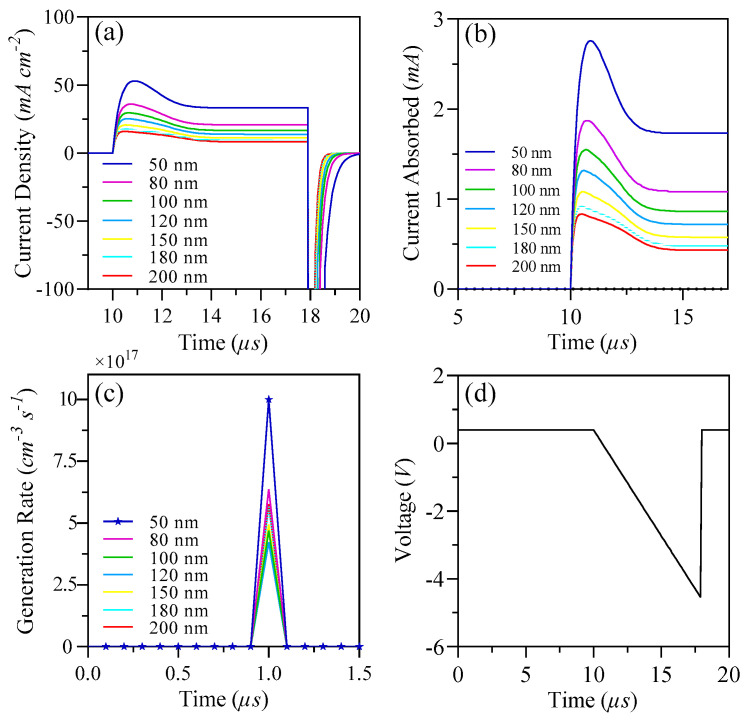
(**a**) Current density of S1; (**b**) current absorbed for S1; (**c**) generation rate for S1; and (**d**) voltage for S1.

The assessment of charge extraction is performed utilizing the photo-CELIV technique. The results of these measurements are illustrated in Figure 13. Specifically, Figure 13a,b present the transient photo-current associated with varying mobilities across different thicknesses. Devices that possess a narrower thickness are found to generate higher currents and facilitate quicker charge extraction when compared to their thicker counterparts, particularly those in the 120 nm to 200 nm range. On the other hand, devices with greater thicknesses demonstrate a slight reduction in the generation of current and a slower rate of carrier extraction, as evidenced in Figure 13c. The predominant factor contributing to this observation is the charge recombination rate in thicker devices. As previously indicated, the limited exciton diffusion length in organic materials underscores the importance of optimizing the thickness of the active layer.

At the same time, possessing a wide absorption spectrum is vital for the production of a larger quantity of excitons, while also ensuring efficient diffusion and electron transport at the interfaces. This optimization leads to enhanced charge mobilities and supports rapid charge extraction, ultimately aiding in voltage generation and effective electrode interaction, as shown in Figure 13d.

**Figure 14:**
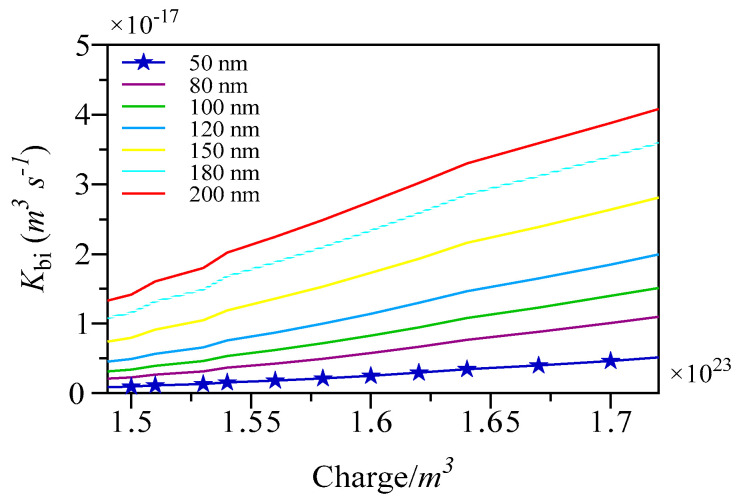
Simulated Q-K_bi_ of S1.

The recombination prefactor (Q-K_bi_) is affected by several factors, such as the specific mechanism of recombination, charge mobility, the presence of an electric field, and the density of states. In our study, Q-K_bi_ for the devices was ascertained through measurements of different active layer thicknesses. A clear correlation was identified between the reduction in the thickness of the active layer and the decrease in the Q-K_bi_, as illustrated in Figure 14. Specifically, the implementation of a 200 nm active layer yielded a high Q-K_bi_ value of 2.7 × 10^−17^ m^−3^ s^−1^ at a charge density of 1.6 × 10^23^ m^−3^. Conversely, a device with a thickness of 50 nm recorded a lower Q-K_bi_ of 0.25 × 10^−17^ m^−3^ s^−1^ under the same charge density conditions.

Further evidence suggests that an increase in thickness may impede the efficiency of charge extraction, disrupt dissociation processes within the active layer, and obstruct charge transport to the electrodes, ultimately detracting from the device’s overall performance.

We compared our results with other works conducted by Rafiq et al. [54], Ram et al. [55], and Zhu et al. [42], as summarized in Table 5. The focus of our research was the simulation of binary OSCs employing SnO_2_ as an ETL. We compared our findings with those from previously mentioned studies. The simulations revealed that single-junction OSCs demonstrated a significantly enhanced PCE relative to the data presented in Table 5. Remarkably, we recorded a J_sc_ of 28.2 mA cm^−2^, which represents the highest J_sc_ documented in theoretical investigations to date. The achievement of an extended absorption wavelength in the EQE through our specific configuration, along with an optimized active layer thickness of 50 nm, was instrumental in enhancing exciton separation and the generation of free charge carriers, thereby leading to an increase in the J_sc_. Furthermore, our ternary device surpassed the performance metrics reported by Ram et al. and Zhu et al., attaining a significantly elevated V_oc_ of 82.3 V. In contrast, the findings of Rafiq et al. indicated that their OSC devices exhibited superior V_oc_ and FF values, recorded at 0.999 V and 88.52%, respectively. Our structural analysis reveals that the higher V_oc_ and FF values observed in Rafiq et al.’s work can be ascribed to their implementation of a triple ETL composed of C_60_, PC_60_BM, and ZnO, which enhances energy alignment between the LUMO of these materials and a cascading energy level system that promotes effective electron transfer to the silver electrode. Despite our OSCs achieving higher V_oc_ and FF values, our optimal PCE of 19.85% surpasses the results documented in the other studies presented in Table 5.

**Figure 15:**
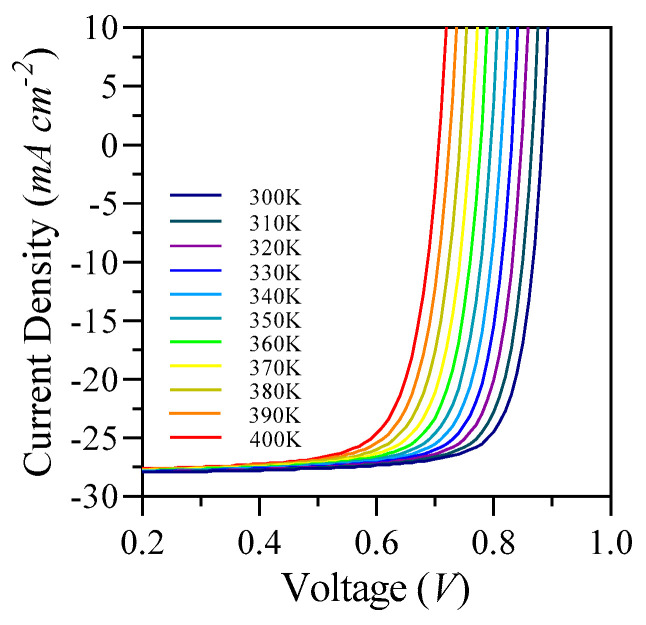
S1 simulated J-V curves.

**Figure 16:**
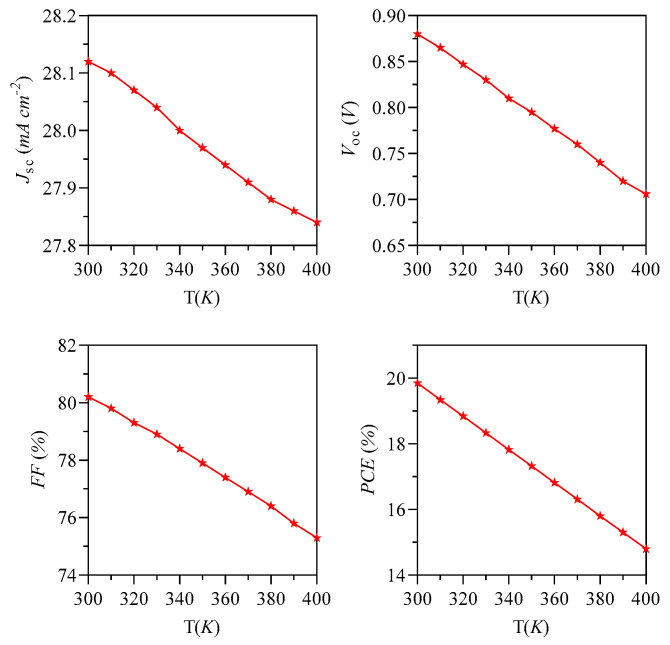
Simulation of the influence of temperature on the performance indicators of the S1: J_sc_, V_oc_, FF and PCE.

The J-V characteristics were simulated under AM 1.5 G illumination at an intensity of 100 mW cm^−2^, across a temperature spectrum of 300 K to 400 K. The material S1 exhibited the highest PCE of 19.85% at 300 K, with a J_sc_ of 28.2 mA cm^−2^, an Voc of 0.88 V, and a FF of 80%. However, as the temperature increased from 310 K to 400 K, the performance declined, resulting in a PCE of 14.80%, a J_sc_ of 27.84 mA cm^−2^, a Voc of 0.71 V, and an FF of 75.3% at 400 K. It is noteworthy that the primary factors contributing to the decrease in OSCs efficiency with rising temperatures are V_oc_ and FF, as illustrated in Figure 15 and Figure 16, along with Table 6.

For this study, we selected highly stable charge transport interface materials, specifically PMMA and SnO_2_, both characterized by high T_g_ and notable hole and electron mobilities. PMMA demonstrated hole and electron mobilities of 1.9 × 10^−6^ m^2^ V^−1^ s^−1^ and 1.0 × 10^−13^ m^2^ V^−1^ s^−1^, respectively, while SnO_2_ exhibited mobilities of 2.0 × 10^−6^ m^2^ V^−1^ s^−1^ for holes and 2.9 × 10^−5^ m^2^ V^−1^ s^−1^ for electrons. These findings are summarized in Table 7. The effective performance observed at elevated temperatures as depicted in Figure 15 and 16 can be attributed to the high mobilities of the ETL and HTL, as well as the T_g_ of these materials, which contribute to the overall stability of the device.

A rise in temperature leads to a proportional enhancement in ΔE, as indicated by Equation (15), which consequently results in a decrease in V_oc_. The FF showed an inverse relationship with temperature, declining as the temperature transitioned from 300 K to 400 K. This decline in FF can be attributed to the exponential increase linked to temperature, as discussed in the analysis. The exponential relationship between temperature and the reverse saturation current in photovoltaic cells is represented in Equation (16). Furthermore, this variable may also have effects on the J_sc_.

## 4. Conclusions

The study presents a comprehensive analysis of optoelectronic simulations conducted on devices featuring a p-i-n binary OSC architecture, specifically utilizing layers of ITO/PMMA/PM6:D18:L8-BO/ETL/Ag. The research focuses on two configurations with SnO_2_ and ZnO as ETLs. The most effective device, utilizing SnO_2_, achieved a PCE of 19.85%, with an optimized active layer thickness of 50 nm. This configuration yielded a V_oc_ of 0.88 V, a J_sc_ of 28.2 mA cm^−^^2^, and an FF of 80%. Notably, the performance of the SnO_2_-based device was found to be superior to that of the ZnO-based device, which is attributed to the advantageous crystalline structure of SnO_2_. The SnO_2_-based device demonstrated a light absorption efficiency of 90% across a broad visible wavelength range of 380–880 nm, significantly improving charge carrier mobility and minimizing energy losses. The findings indicate that the J_sc_ of 28.2 mA cm^−^^2^ surpasses the previous literature, highlighting the importance of selecting compatible active and transport materials. Additionally, the study reports a minimal ΔV_oc_ of 0.4 V and maintains a robust efficiency of SnO_2_-based OSCs under elevated temperatures, with less than a 12% decrease in efficiency at 350 K compared to room temperature. Future research directions emphasize the need for a holistic approach to enhancing OSC configurations, aiming to reach the theoretical Shockley–Queisser limit for single-junction OSCs. This includes exploring optical engineering techniques to improve light absorption, conducting detailed electronic and mathematical analyses to understand the physical characteristics of the cells, and investigating advanced materials and microstructural designs to further enhance efficiency. Furthermore, the creation of a solar cell device grounded in the optimal parameters obtained from the simulations would represent a significant approach to affirming the results and bolstering the paper’s contributions to the field.

## Figures and Tables

**Table 1:** Thicknesses of SiO_2_-based OSCs materials.

Layer Name	Thickness (nm)	Optical Material	Layer Type
ITO	50	Oxides	Contact
PMMA	35	Polymers	HTL
D18:L8-BO	50	BHJ	Active
SnO_2_	45	Oxides	ETL
Ag	100	Metal	Contact

**Table 2:** Thicknesses of ZnO-based OSCs materials.

Layer Name	Thickness (nm)	Optical Material	Layer Type
ITO	50	Oxides	Contact
PMMA	35	Polymers	HTL
D18:L8-BO	50	BHJ	Active
ZnO	45	Oxides	ETL
Ag	100	Metal	Contact

**Table 3:** Input electrical parameters of the simulation.

Parameters	Values
Free electron’s Effective density (N_c_ at 300 K)	1 × 10^26^ m^−3^
Free hole’s Effective density (N_v_ at 300 K)	1 × 10^26^ m^−3^
Electron mobility (μ*_e_*)	1.4 × 10^−7^ m^−2^V^−1^s^−1^
Hole mobility (μ_h_)	1.8 × 10^−7^ m^−2^V^−1^s^−1^
Trapped electron (n_trap_) to free hole (P)	1 × 10^−20^ m^−2^
Trapped hole (P_trap_) to free electron (n)	1 × 10^−20^ m^−2^
n to P recombination rate constant	7.86 × 10^−17^ m^−3^s^−1^
Free electron (n) to trapped electron (strap)	1 × 10^−15^ m^−2^
Free hole (P) to trapped hole (P_trap_)	1 × 10^−15^ m^−2^
Energy bandgap (E_g_)	1.32 eV
Relative permittivity (Ɛ_r_)	3.0 a.u
Number of traps (N_t_)	5 Traps

**Table 4:** The PCE parameters output of each structure with different active layer thicknesses for S1 and S2.

Devices	Thickness (nm)	V_oc_ (v)	J_sc_ (mA cm^−2^)	FF (%)	PCE (%)
S1: ITO/PMMA/D18:L8-BO/SnO_2_/Ag	50	0.88	28.2	80	19.85
	80	0.87 (0.869 ± 0.001)	25.34 (25.30 ± 0.04)	78 (78.8 ± 0.2)	17.07 (17.05 ± 0.02)
	100	0.86 (0.858 ± 0.002)	23.52 (23.02 ± 0.05)	75 (74.9 ± 0.1)	15.26 (15.25 ± 0.01)
	120	0.855 (0.858 ± 0.002)	23.28 (23.25 ± 0.03)	73 (72.9 ± 0.1)	14.47 (14.45 ± 0.02)
	150	0.85 (0.849 ± 0.001)	24.61 (24.60 ± 0.01)	68 (67.7 ± 0.3)	14.20 (14.19 ± 0.01)
	180	0.847(0.84 ± 0.007)	25.71 (25.70 ± 0.01)	62.5 (61.4 ± 0.1)	13.62 (13.60 ± 0.02)
	200	0.845 (0.84 ± 0.005)	26.45 (26.40 ± 0.05)	59 (58.7 ± 0.3)	13.09 (13.05 ± 0.04)
S2: ITO/PMMA/D18:L8-BO/ZnO/Ag	50	0.88	27.7	80	19.60
	80	0.867 (0.868 ± 0.001)	25 (25.10 ± 0.1)	77.5 (77 ± 0.5)	16.84 (16.80 ± 0.04)
	100	0.86 (0.855 ± 0.001)	23.34 (23.30 ± 0.04)	75.3 (75.2 ± 0.1)	15.13 (15.11 ± 0.02)
	120	0.855 (0.854 ± 0.001)	23.22 (23.20 ± 0.02)	72.6 (72.5 ± 0.1)	14.42 (14.40 ± 0.02)
	150	0.85 (0.848 ± 0.002)	24.60 (24.00 ± 0.6)	67.7 (67.5 ± 0.2)	14.18 (14.14 ± 0.04)
	180	0.847 (0.845 ± 0.002)	25.70 (25.5 ± 0.2)	62.5 (62.4 ± 0.1)	13.60 (13.55 ± 0.5)
	200	0.845 (0.843 ± 0.002)	26.40 (26.30 ± 0.1)	58.6 (58.5 ± 0.1)	13.07 (13.05 ± 0.02)

**Table 5:** Comparison between our work and other theoretical p-i-n OSCs.

Reference	Structure	V_oc_ (v)	J_sc_ (mA cm^−2^)	FF (%)	PCE (%)
Zhu et al. [42]	ITO/PEDOT:PSS/PM6:D18:L8-BO/PNDIT-F3N/Ag	0.87	24.49	80.38	17.21%
Rafiq et al. [54]	ITO/MoO_3_/PDTS-DTTFBT:PC71BM/C_60_/PC_60_BM/ZnO/Ag	0.999	20.01	88.52	17.69%
Ram et al. [55]	ITO/WS_2_/PBDB-T-2F:Y6:PC_71_BM/PFN-Br/Al	0.85	25.1	80	17.10%
Boudia et al. [18] (our previous work)	ITO/PEDOT:PSS/PM6:L8-BO/SnO_2_/Ag	0.859	26.5	80.4	18.34%
Our work	ITO/PMMA/D18:L8-BO/SnO_2_/Ag	0.88	28.2	80	19.85

**Table 6:** Simulated performance indicators of S1.

	T(K)	V_oc_ (v)	J_sc_(mA cm^−2^)	FF(%)	PCE(%)
S1	300	0.88	28.12	80.2	19.85
	310	0.865 (0.864 ± 0.001)	28.10 (27.90 ± 0.2)	79.8 (79.7 ± 0.1)	19.34 (19.30 ± 0.04)
	320	0.847 (0.845 ± 0.002)	28.07 (28.05 ± 0.2)	79.3 (79.1 ± 0.2)	18.84 (18.79 ± 0.05)
	330	0.83 (0.828 ± 0.002)	28.04 (28.03 ± 0.1)	78.9 (78.6 ± 0.3)	18.33 (18.33 ± 0.04)
	340	0.81 (0.805 ± 0.006)	28 (27.90 ± 0.1)	78.4 (78.1 ± 0.3)	17.82 (17.77 ± 0.05)
	350	0.795 (0.793 ± 0.002)	27.97 (27.96 ± 0.1)	77.9 (77.8 ± 0.1)	17.32 (17.30 ± 0.02)
	360	0.777 (0.775 ± 0.002)	27.94 (27.92 ± 0.2)	77.4 (77.2 ± 0.2)	16.81 (16.80 ± 0.01)
	370	0.76 (0.755 ± 0.005)	27.91 (27.90 ± 0.1)	76.9 (76.8 ± 0.1)	16.31 (16.30 ± 0.01)
	380	0.74 (0.735 ± 0.005)	27.88 (27.85 ± 0.3)	76.4 (76.3 ± 0.1)	15.80 (15.79 ± 0.01)
	390	0.72 (0.715 ± 0.005)	27.86 (27.85 ± 0.1)	75.8 (75.7 ± 0.1)	15.30 (15.31 ± 0.01)
	400	0.706 (0.70 ± 0.006)	27.84 (27.81 ± 0.3)	75.3 (75.2 ± 0.1)	14.80 (14.79 ± 0.01)

**Table 7:** The glass transition temperature (Tg) and charge mobilities of the PMMA and SnO_2_.

Materials	Tg or Tm (K)	Ref.	Holes Mobility (m^2^ v^−1^ s^−1^)	Ref.	Electron Mobility (m^2^ v^−1^ s^−1^)	Ref.
PMMA	401	[56]	1.9 × 10^−6^	[57]	1.0 × 10^−13^	[58]
SnO_2_	1903	[59]	7.6 × 10^−4^	[60]	2.6 × 10^−2^	[60]

## Data Availability

The data are available upon reasonable request from the corresponding author.
